# Beyond the limits of clinical governance? The case of mental health in English primary care

**DOI:** 10.1186/1472-6963-8-63

**Published:** 2008-03-26

**Authors:** Linda Gask, Anne Rogers, Stephen Campbell, Rod Sheaff

**Affiliations:** 1 National Primary Care Research and Development Centre, University of Manchester, Williamson Building, Oxford Road, Manchester M13 9DL, UK; 2 University of Plymouth, Drake Circus, Plymouth, Devon PL4 8AA, UK

## Abstract

**Background:**

Little research attention has been given to attempts to implement organisational initiatives to improve quality of care for mental health care, where there is a high level of indeterminacy and clinical judgements are often contestable. This paper explores recent efforts made at an organisational level in England to improve the quality of primary care for people with mental health problems through the new institutional processes of 'clinical governance'.

**Methods:**

Framework analysis, based on the Normalisation Process Model (NPM), of attempts over a five year period to develop clinical governance for primary mental health services in Primary Care Trusts (PCTs). The data come from a longitudinal qualitative multiple case-study approach in a purposive sample of 12 PCTs, chosen to reflect a maximum variety of organisational contexts for mental health care provision.

**Results:**

The constant change within the English NHS provided a difficult context in which to attempt to implement 'clinical governance' or, indeed, to reconstruct primary mental health care. In the absence of clear evidence or direct guidance about what 'primary mental health care' should be, and a lack of actors with the power or skills to set about realising it, the actors in 'clinical governance' had little shared knowledge or understanding of their role in improving the quality of mental health care. There was a lack of ownership of 'mental health' as an integral, normalised part of primary care.

**Conclusion:**

Despite some achievements in regard to monitoring and standardisation of prescribing practice, mental health care in primary care seems to have so far largely eluded the gaze of 'clinical governance'. Clinical governance in English primary mental health care has not yet become normalised. We make some policy recommendations which we consider would assist in the process normalisation and suggest other contexts to which our findings might apply.

## Background

One specific approach in the international 'quest for quality' in health care has been a standardization of practices in medicine. It began early in the twentieth century, but gathered speed with the emerging discourse of 'Evidence-Based Medicine' in the 1990's [1]. Most attention has been given to the development and operationalisation of practice and clinical guidelines, which assemble evidence from scientific research into particular recommendations for health practitioners [2]. Less attention has been given to the organisational as opposed to the technical aspects of implementation or on implementation in clinical areas such as mental health where there is a high level of indeterminacy and contestability (i.e. the tacit private and less technical aspects of clinical judgement). In primary care (a domain into which much clinical work is now being re-directed [3] there is also evidence that tacit rather than explicit research based knowledge underpins much professional work [4]. This paper explores organisational-level efforts to improve the quality of care for people with mental health problems in primary care. Although the data come from the English National Health Service (NHS), the organisation of primary mental health care there is sufficiently similar to certain other health systems and to the provision of care for other care groups (see below) to give the findings wider relevance.

### Clinical governance

The policy of 'clinical governance' gives English NHS organisations a statutory duty to make arrangements for monitoring and improving the quality of care that they provide. Its purpose is described as being:

*"To assure and improve clinical standards at local level throughout the NHS. This includes action to ensure risks are avoided, adverse events are rapidly detected, openly investigated and lessons learned, good practice is rapidly disseminated and systems are in place to ensure continuous improvements in clinical care*[5]*."*

Clinical governance is concerned with the work involved in both getting both quality assurance and improvement integrated into routine everyday practice in health care [6]. Implementing this agenda has been a huge management task, not just because of the infrastructure it requires, but also because of the cultural changes it engenders [7] and its reliance on relatively unfamiliar forms of professional self-regulation. Clinical governance policy are also rests on the rather managerialist assumption that senior NHS managers are able to ensure that clinical governance activities occur within the organisations under their management, and are responsible for doing so.

The National Institute for Health and Clinical Excellence (NICE) and the Commission for Health Audit and Improvement (CHAI; now the Healthcare Commission) provide detailed guidance about the quality of health care and processes for achieving it [8,9]. For specific care groups, national standards have been set through National Service Frameworks (NSFs). These standards stipulate both clinical standards and the (organisational) models of care into which clinical practice is embedded. For example, standard two of the Mental Health NSF [10] stipulates:

'Any service user who contacts their primary health care team with a common mental health problem should:

• *have their mental health needs identified and assessed*

• *be offered effective treatments, including referral to specialist services for further assessment, treatment and care if they require it* (p.28)*.'*

Six other standards concern mental health promotion, access to services, 'Caring about Carers' and preventing suicide. Each standard is elaborated with a set of aims, description of interventions and their evidence base, care models and examples of good practice, and implementation 'milestones'. Although Mental Health was the first NSF, NHS bodies have generally afforded that particular NSF lower status and priority than those for other conditions such as coronary heart disease published afterwards.

### Bringing clinical governance into primary mental health care

In English primary care clinical governance and the implementation of NSFs are constrained by the fact that general practitioners (GPs) mostly remain independent contractors not NHS employees. In English Primary Care Trusts (PCTs), clinical governance is therefore accomplished through local medical networks under the local professional leaders whom doctors recognise, rather than general managers, as their 'politically' legitimate leaders:

*'Some English GPs now exercise a soft governance over others through a gradual introduction of managerial techniques and rather subtle individual incentives, the latter being more moral rather than material *[11]* (*p425)*.'*

As in many other countries, NHS primary mental health care itself is also largely delivered through local networks of staff who are employed by different organisations but, at least in theory, operate agreed common care pathways. Degeling and colleagues [9] note that clinical governance policy, and the way it is implemented, fails to consider how to improve specific care pathways for commonly occurring problems, which is what would make the concept of clinical governance more meaningful for clinical staff. The rather managerialist conception of 'clinical governance' described above is thus at odds with the way in which most mental health care is actually delivered. Consequently implementation of the mental health NSF was reportedly more organizationally complex than implementing the coronary heart disease NSF, with PCTs being less willing to dedicate resources to it [12].

In this study we therefore attempt to understand more about why the clinical governance of mental health care in primary care (as Rogers et al. [12] suggest) seems to pose a particular challenge to those wishing to normalise the quality improvement initiatives which clinical governance involves. (Here, 'normalisation' means 'the embedding of a technique, technology or organizational change as a routine and taken-for-granted element of clinical practice' [13 p2]. Given the organisational complexity of NHS primary mental health care, what do the different actors do, or perceive that they need to do, to normalise clinical governance activity in that domain?

## Methods

To examine the extent to which clinical governance of mental health care has been normalised within NHS primary care we used a framework analysis [13]. We adopted the Normalisation Process Model (NPM) [14-16] rather than one of the many other conceptualisations of the adoption of innovations and of knowledge management because normalising evidence-based practice is the central aim of clinical governance policy in the English NHS. This analytic framework thus enables one to judge what success the implementation of this policy has had, in its own terms, in the sphere of mental health. However the NPM is applicable to the normalisation of new clinical practices in general.

From earlier empirical studies the NPM identifies two pairs of conditions which maximise the likelihood that a complex new working practice will become normalised:

1. Within the clinical encounter:

*(a) Interactional workability *i.e. whether the new working practice is consistent with clinicians and patients sharing assumptions about what clinical work should be done, its legitimacy, its goals, meaning, outcomes and the legitimate forms of conduct and cooperation of each party. For example, the use of digital cameras for on-line dermatological diagnosis only weakly satisfies the interactional workability condition, for that practice focuses the clinician-patient interaction on the camera and a computer-aided protocol rather than on the direct patient-clinician interaction [14].

*(b) Relational integration *i.e. whether the new working practice embodies what clinicians personally regard as valid (clinical) knowledge, as appropriate expertise, and the appropriate sources of that expertise; and how far the new working practice conforms to existing public assumptions about what knowledge is credible, useful and authoritative. For example, video-conferenced psychiatric consultations had weak relational integration because they reduced the certainty of interpretation of patients' expressed symptoms and responses [14].

2. Two conditions which concern the organisational setting:

*(a) Skill-set workability *i.e. whether the new working practice is compatible with the existing division of clinical labour, methods of monitoring clinical work, allocation of resources and rewards, competence boundaries, degree of clinical autonomy expected for practitioners, and the expected quality of their work. For instance nurse-led home telecare for people with COPD strongly satisfies this condition because it fits well with specialists nurses' existing activities [14].

*(b) Contextual integration *i.e. 'the capacity of ... [the host] organization to understand and agree the allocation of control and infrastructure resources to implementing a complex intervention, and to negotiating its integration into existing patterns of activity' [14]. Remote diagnosis for non-urgent dermatological conditions, for example, only weakly satisfies this condition because it makes the funding, organisation and delivery of specialist clinics more complicated and increases specialists' workloads [14].

Determining how far clinical governance empirically satisfies these four (sets of) conditions would enable one to judge how far and through what processes clinical governance has already become normalised in English primary mental health care, or is likely to.

Clinical governance is essentially a set of organisational processes for the review and revision of substantive clinical working practices. Consequently the two clinical-level conditions for normalisation would barely apply to it. According to NPM, clinical governance activities would only need to have high interactional workability (in order to become normalised) to the extent that they became part of the clinician-patient relationship itself. But it is hard to conceive of ways in which clinical governance activities themselves would do so directly. Rather, they would at most influence that relationship indirectly through whatever changes in substantive clinical working practices they promulgated. Insofar as clinical governance activity has (or needs) the property of interactional workability at all, that property is therefore derivative from the interactional workability of the substantive clinical practices which clinical governance activity promulgates. Similarly, relational integration is a property of the substantive working practices which clinical governance activity might promulgate rather than of the organisation processes of clinical governance themselves. Again, the latter would acquire the property of relation integration indirectly and derivatively from whatever substantive clinical working practices were promulgated through clinical governance activity. In any event, it is beyond the scope of a single paper to assess, severally, the interactional workability and relational integration of all these working practices. The present framework analysis therefore notes these two conditions only in the exceptional cases when they (too) appeared from the data. It concentrates on the two sets of organisational conditions which the NPM mentions: skill-set workability and contextual integration.

To populate those parts of an NPM-based analytic framework, this study draws on a programme of research carried out over a five year period into the implementation of clinical governance in Primary Care Trusts (PCTs) in England. It employed a longitudinal qualitative multiple case-study approach in a purposive sample of 12 organisations, chosen to reflect a maximum variety of organisational contexts for mental health care provision. We selected case study sites to include three PCTs that directly provided mental health care, and others which did not; and PCTs which ranged from 'Beacon' sites to sites reported to be having difficulty providing mental health services. Data collection focussed on the implementation of two of the first National Service Frameworks (NSFs) to be introduced in England i.e. for mental health [10] and coronary heart disease [17] as concrete exemplars that could be compared across sites. Initial data collection took place during September to November 2000, involving semi-structured interviews with key informants including the chief executive, clinical governance lead, mental health lead, and a lay informant. Other aspects of the implementation of clinical governance in these 12 case study sites on the basis of this first wave of interviews have been reported elsewhere [7].

We revisited these Primary Care Trusts during 2003–4, to carry out further interviews with clinical governance leads and managers (12 interviews carried out with 17 informants), audit leads (3 interviews) and mental health leads (11 interviews with 18 informants- one site could not identify a lead) to explore how implementation of clinical governance had progressed. Additionally we interviewed informants identified as by PCT informants as 'primary care' leads at the local Mental Health provider trust. For three of the sites, as the Trust was an integrated provider of primary care and mental health, there was no need to conduct a further interview. A further site arranged a single group interview with four representatives from both primary care and mental health. Four interim interviews with mental health leads in both PCTs and mental health trusts were carried out at two sites during 2001, selected on the basis of the case study profiles because they might provide contrasting views of the development of primary care mental health provision. The mental health leads at these sites were unchanged from the previous year, however by 2003–4 the PCT mental health leads had changed in all but 3 of the 11 sites for which we could identify a lead person. A total of 41 interviews carried out with 49 informants form the main empirical material for the analysis. The anonymity of all interviewees was assured.

The 41 new interviews were initially coded using MaxQDA qualitative software. Emergent themes were discussed between the authors and throughout the process of analysis, these themes were defined, focused and altered. Earlier material from the first wave of interviewing was utilised both to triangulate findings and explore any changes that had occurred. All quotations in the text are from 2003–4 interviews unless otherwise stated.

## Results and Discussion

Besides 'clinical governance', something called 'primary mental health care' (whose definition differed from site to site) emerged from the data as a second related 'object' or complex intervention which was also to be implemented. We therefore treat them together when comparing them with the two sets of organisational conditions which the NPM framework assumes promote normalisation.

### Skill set workability

#### a) Creating 'primary mental health care'

In policy terms the notion of 'primary mental health care' had emerged against a background and skill set in which secondary care norms and modes of operation dominated the consciousness and practice of mental health care and everyday clinical practice [18]. There was considerable variation in the extent to which general practitioners felt able or prepared to work with people with mental health problems and probably a greater variation in threshold for referral [19] for mental ill-health than for any other presenting health problem:

*'they may be a perfectly competent GP but they just haven't got that level of training to know that, okay, that person may calm down if you just spend a bit longer with them, they maybe haven't got that level of skill, maybe they're frightened, may be they just don't want to do it, it's a mixture of things. Its often a feeling of I can't cope with this because mental health problems, a lot of them are very messy, they're not clear-cut*.' (Site F PCT mental health lead (GP))

So it is not surprising that our informants reported a gap in service provision, a *'grey area' *between what was provided for people with mental health problems in general practice, and by Community Mental Health Teams in mental health trusts which have, in recent years, focussed much more on 'severe and enduring mental illness':

*'So we have a cohort of patients- service users who are too severe for the counselling service, don't meet the criteria for the CMHT specialist services and GPs are finding they don't quite know what to do with these patients...So some of our GPs have had training and they're very interested and they can do short-term interventions but the vast majority of GPs out there wouldn't have a clue.' *[Site H mental health lead PCT (commissioner)]

How sites were responding to this problem seemed to depend on a number of factors. Dissatisfaction expressed by the GPs about *'the number of patients who were either, if you like, bounced back from secondary care, or, simply just not taken on by secondary care at all' *[Site E combined PCT/Mental Health Trust mental health manager] was clearly an important factor, as was the discontent caused by service inequities across the PCT. But the nature of the response and the sense of ownership of this problem to challenge working practices within primary care itself rather than the practices of the specialist mental health sector varied from site to site. It seemed either to depend historically on the presence of key actors in primary care, or within the mental health trust, who had interest in developing something that they called 'primary care mental health services' or the way in which the service had been historically configured.

At PCT level, some actors emerged as both formal and informal leaders in commissioning mental health services, particularly where they shared a common provider mental health trust and had managed to maintain some continuity of senior staff:

Mental health lead 1: *'I think just by sheer default we will almost be the leaders at this stage because we seem slightly more organised, which I hesitate to say.'*

Mental health lead 2: *'Yes. I mean I wouldn't say that *[co-provided PCTs]*would agree that we were leading the process... what I would say to you is that the continuity of *[site J]*enables the corporateness to be able to be developed and maintained.' *(Site J)

However, the PCT-level commissioners did not necessarily perceive that they had to possess any 'expert knowledge' to become involved in decision making about mental health care:

*'Oh mental health's a learning curve..... I mean I've been in health since 1990 and it's been in primary care finance you know initially... But I asked to do mental health about a year ago as a development issue and I'm just, I'm falling in love with the subject you know, I think it's really, really interesting.' *[Site H mental health lead 2001]

This interviewee had moved on again by the time of our final interviews in 2003–4. In the absence of clear evidence or direct guidance about what 'primary mental health care' should be, or actors with the power and/or skills to set about realising or executing it, there was a sense of confusion and lack of direction.

#### b) Under-defined roles

Some breaking down of barriers between primary and specialist care had been achieved in all sites with the production of joint guidelines for common mental health problems such as anxiety and depression specified by the NSF as '*must do's'*. It was generally viewed as a success if they had been distributed:

*' there's this sort of glossy package if you will, and that should have gone to all the GPs ... certainly I've got a copy, the teams have got copies of them as well, so as far as I was aware that's been circulated.' *[Site F service manager mental health trust]

But there was recognition that distribution did not equate with implementation:

*'...actually knowing whether the protocols are being followed and practices used, whether they're helpful, I mean the whole evaluation is going to be a nightmare. I have to say that. I acknowledge it's going to be a nightmare. I've chosen not to jump in ...as yet.'* [Site B mental health lead commissioner)]

The actors in 'clinical governance' however had little shared knowledge or understanding of their role in improving the quality of mental health care within the PCTs. So far as monitoring the quality of care was concerned, either they perceived mental health data as being collectable, but only at a basic level, because of its poor quality and inherent complexity, or beyond their remit.

*'I don't think Mental Health is as easy to do as Coronary Heart Disease, we have approached it in terms of developing care pathways, and we have a Mental Health Lead as we have a CHD [coronary heart disease] Lead, so we do work in reaching the targets for the NSF and we have .. organised auditing, it seems to have a lower profile, yeah*.

Q: Why?

*A: Perhaps because it feels not a life and death situation, it could be..*.

*Q: Yes*.

*A: ... it could possibly be, off the top of my head, umm we've certainly got clinicians out there who are very interested in it, that's excellent and we know that standards are improving because of that, at least it... people are more informed about it, that might be a better way of doing that,..... it could well be that there's a lot going on and it's just not an area that I've got involved with .. highly; I know that we get our regular reports on the NSF and that we're meeting our targets there, I know that we've got future developments coming in through our local delivery plan which is about enhancing Mental Health, so I'm confident that there's work going on and some good stuff but, I couldn't write an essay on it.' *[Site H clinical governance lead]

This was not a job that she perceived as being allocated for her to do or that she possessed the skills to perform. However this quote also raises the point that mental health issues are not perceived here as 'life or death' situations. Given the potential for both harm to self and others, an agenda which has been powerful in the development of the 'public safety' agenda in mental health policy, mental health problems clearly are, if not commonly, potentially matters of life and death. Rather, her remarks suggest that she -and possibly other key actors in the organisation – attached relatively low urgency or importance to issues of quality of mental health care. The only data that was routinely utilized in primary care organisation for the purposes of governance was the (relatively easily collected in UK primary care) prescribing data which was subject to scrutiny by the prescribing advisors at the Primary Care Trusts (see below).

#### c) GPs: discretion versus normalisation

We did not collect data from doctors (other than mental health leads) or patients, but the relative importance attributed to primary mental health care within hierarchies of knowledge and practice was apparent:

*'what we did recently was a training needs analysis and the data that was pulled off from there, I don't think it even mentioned Mental Health... Mental Health came about eighth, most of them were about .. neurological conditions, that GPs felt unfamiliar with.' *[Site H Clinical Governance Lead]

Sometimes the problem seemed to extend further- to any work involving people with mental health problems even if it was physical health care. At site J there had been discussion about implementation of the NICE guidelines for Schizophrenia:

*"GPs say well, you know, 'do physical health checks on people with schizophrenia' ... we haven't got the capacity to do that, if they're in secondary services...*.

Q: Did that surprise you?

*A: Not in the slightest." *[Site J mental health trust manager]

GPs and other primary care staff, at all of the sites we visited, were able to exercise a considerable amount of discretion as to whether they engaged in clinical governance activities that related to mental health or not. It was perceived as something that they did not have to do and might not necessarily choose to do given the option. For them, there appeared still to be a failure of normalisation of mental health care in routine primary care. In that case, the creation of 'primary mental health care' within a primary care organisation will challenge basic assumptions about how professionals within the system should relate to each other (relational integration) but also how doctors and patients should negotiate the management of mental health care within the wide range of problems that might be encountered in the primary care consultation (interactional workability).

GPs often have low expectations about whether it is possible for they themselves to intervene effectively with mental health problems [20,21]. Paradoxically, they can be remarkably positive about the impact of counselling despite having limited knowledge of the training and supervision arrangements which are necessary for the safe employment of counsellors in primary care [22]. This apparent conflict is probably best understood in terms of the desire of many GPs (particularly those with a limited knowledge or interest in mental health) to pass these problems onto another professional, perhaps with an assumption that, if nothing much seems to have a positive impact for people with mental health problems, then nothing much can do any harm? Whatever the beliefs or assumptions of the GPs, we certainly identified a clear preference for the 'referral' route the management of mental health problems which meant GPs neither needed to see themselves as, nor act as, the key actors in mental health. Rather they considered that through the mechanism of referral, the major responsibility lay within the secondary care sector, although the threshold for referral undoubtedly varies between doctors [19]. This lack of ownership not only contributed to the failure of implementation of 'clinical governance' in relation to mental health but also to the problematic progress in the construction of 'primary mental health care' within the organisations and in its normalisation. Insofar as the skill-set workability of mental health care was implicitly considered at all, its skill-set workability was implicitly rated low by GPs.

#### d) Polar experiences of skill set workability

Two contrasting levels of skill set workability were instantiated by the introduction of graduate mental health workers and by prescribing. The problematic implementation of new primary care mental health workers called 'graduate mental health workers' over the last five years in England [23] highlighted the different responses, and sometimes resistance, to the normalisation of a new working practice when the ownership of knowledge, work-flows and inter-professional relationships were already unclear even before the new workers were introduced to the system [24]. An opposite example was that the monitoring and standardisation of prescribing practice proved relatively easy to achieve. The requisite data were already collected in detail from pharmacists for re-imbursement purposes, and included data on drugs used for mental health care. By the time of this study it was already established routine practice for these data to be fed back to PCTs, to the collective body of GPs within the PCT, and to the individual prescribers. (This also represents a high level of relational integration.) In all sites, each prescriber knows how to identify his or her own prescribing patterns and compare them with local averages. In some (not all) study sites, the data were not even anonymised, so that each prescriber could also know who was adopting which prescribing patterns. It was a relatively straightforward step to make prescribing reviews a routine and central clinical governance activity.

### Contextual Integration

#### a) Change, fragmentation, re-integration

The most striking feature of the organisations that we studied was their persistent instability of organisational structures, identity, personnel and strategic direction. The last five years have seen major, barely interrupted changes in the configuration of health authorities and trusts in England. This meant that some of the emerging organisations that we visited in 2000 were, by 2003–4, part of larger new PCTs serving more than twice the original population. Ostensibly these mergers were aimed at increasing efficiency and improving integration, although such centralisation of purpose has sometimes proved difficult to achieve in practice [25]. The three PCTs that directly provided mental health care had remained relatively unchanged during 2000–2003 but the configuration of the mental health provider trusts for the other sites had changed radically. Across the sites as a whole, few of the people that we interviewed in 2003–4 were the same as in 2000 even though in 2003–4 we interviewed people who held the same, or nearest equivalent, roles to our 2000 interviewees. These changes impeded the contextual integration of clinical governance, and the NSF, in primary mental health care.

#### b) Targets, tick-boxes- and a policy vacuum

In 2000 we found that there was general optimism about the National Service Framework (NSF) and the impact that it might have:

*'So far we are pleased that the way forward has been dictated by the National Service Framework....the NSF has given at least a benchmark to various statutory authorities that they've got to deliver and it is prescriptive and it is quite clear what we need to do.' *(Site K)

However it soon became apparent that there was a lack of specificity in the NSF about the nature of 'primary mental health care' to be commissioned, compounded in the NHS Plan [26] despite the inclusion of several specific targets for specialist mental health services:

*'In terms of specifically primary care I would...say we haven't done as much as we might have liked, because the priorities around the NSF milestones have been the assertive outreach and crisis resolution which are more at the end of the severe and enduring end of things.... you can also say, to be brutally honest about it, that the key things that matter are the, the targets, and, you know, you could sort of say the rest, you could if you were really hard-nosed, say "Well, you know, if this doesn't meet the target, let's just forget about it.' *(Site C PCT mental health lead)

When the NSF arrived in 1999, primary care organisations were clearly at different levels of preparedness to implement it. For some, the NSF acted as a challenge to act and reinforce an ongoing creative process of negotiation between actors about the formal knowledge and practice of something called 'primary mental health care'. However, for others, where such conversations had not yet begun, or were stalled, progress was much slower.

'Mental health' seemed to be something that the PCT mental health lead did only in terms of 'developing services', often with no specific way of ensuring that they were either based on best evidence or evaluated as an integral part of the commissioning and/or development process. This fits with the view of mental health service development as '*an image of heroic pirates resourcefully bending the rules' *[27] (p.68). The disconnection between the formal managerial 'commissioning' view and the actual work going into developing the service through informal networks in the organisation was most striking at site F:

*'I certainly found coming in as mental health lead I sort of didn't quite know what the joint commissioning team were doing, I knew they were doing lots of stuff but it didn't feel connected at all...I've tried to draw people in before, people would perhaps come to a meeting or two but because it wasn't their main remit and in a sense it wasn't someone with sufficient seniority really to get in there and make sure its included in the strategic thinking of the PCT.' *[site F mental health lead (GP)]

#### c) Gaps between primary and secondary care

Paradoxically, where the PCT itself provided integrated primary care and mental health services (3 sites) there seemed little sense that 'mental health care' should be owned by or fostered within the primary care wing of the organisation, indicating an apparent absence of actual integration of working practices within these organisations. In one of these PCTs there was not even regular primary care input into the Local Implementation Team, the local group tasked with steering implementation of the NSF *'I'll be honest I've never thought of inviting the Primary Care Commissioning Manager.' *[Site G combined PCT/Mental Health Trust mental health lead]

Despite the key position of mental health trusts there was remarkably little joint activity that could be construed as clinical governance across the interface between primary and secondary mental health care other than infrequent joint educational or liaison events. More intensive links were a minority and tended to be focussed around a particular project or '*piece of work' *like the development of a protocol. There was limited recognition that such activities might, like service development, be considered to be the part of the ongoing work of clinical governance of mental health care.

## Conclusion

The NPM predicts that in order to become normalised, new working practices such as clinical governance activities have to satisfy four (sets of) conditions. The contested nature and status of 'mental health' within primary medical care makes it particularly difficult to change clinical working practices and the ways in which patients and professionals themselves interact, i.e. to satisfy the interactional workability and relational integration, insofar as they apply to clinical governance activities. It also compounds the (more substantial) skill-set and contextual problems and uncertainties faced by those who seek to 'improve the quality of primary mental health care'. The data show a lack of clear conceptualisation about what primary mental health care is or ought to be, under-defined roles and wide professional discretion, especially for GPs. They also suggest that clinical governance and the mental health NSF only weakly satisfy the NPM's contextual integration conditions. This is not for want of willingness on senior managers' or clinicians' parts but more due to lack of knowledge about what (material and human) resources are required and how they can be used to integrate clinical governance activity, including NSF implementation, more centrally into mental health care; and how to start bridging the service gaps noted above.

In England, the benefits of the clinical governance policy for primary mental health care have so far included improved basic monitoring and standardisation of prescribing practice – which are not trivial achievements; and greater managerial salience for primary mental heath care with a recent notable attention to the workforce requirement to implement improved access to psychological therapies in this setting which post-dated this research. Its main shortcomings, described above, stem largely from long-standing neglect of primary mental health care by policy-makers and managers, and the lack of clinicians (of all disciplines) whose prime role is explicitly to fill the service gaps noted above. Clinical governance in English primary mental health care has therefore had to develop from a relatively low base. On balance, primary mental health care seems to have largely eluded the gaze of 'clinical governance'; clinical governance is far from normalised yet.

Taken with the NPM framework, the above data nevertheless also imply that clinical governance in primary mental healthcare could in principle yet meet the two organisational conditions for normalisation. In order to normalise 'clinical governance in mental health' in primary care settings, the work of primary care mental health' (which emerged as a second 'object' in the analysis) must also be normalised. There are thus two related processes at play here: that of the normalisation of 'clinical governance' but also the process of normalisation of 'primary mental health care',

An obvious policy recommendation which flows from these findings is to develop and then evaluate new models of care designed to bridge the gaps between primary and secondary mental health care. In doing so, the uncertainties about what skill-sets are required in primary mental health care could be reduced. A long-term policy commitment to improve the evidence base of clinical practice in primary mental health care is another obvious policy recommendation (and one often heard). Comparing the above findings with the much greater impact of clinical governance in better-evidenced areas of care such as coronary heart disease [28], suggests that such a policy commitment would probably be the biggest single policy contribution to increasing the impact of clinical governance in primary mental health care. At managerial level, one obvious short-term recommendation is to establish information systems that can monitor patient flows between the primary mental health care providers, and subsequent clinical outcomes, as effectively as prescribing is monitored – and therefore managed. Another, pending the development and evaluation of new models of care, is to ensure that referral criteria and care pathways for people with mild to moderate mental health problems are better-defined.

Various research implications also follow. Although the above findings concern the English NHS, primary mental health care in some other health systems (e.g. Ireland, Australia, New Zealand, the Netherlands besides Northern Ireland, Scotland and Wales) has similar organisational and skill-set characteristics, making the foregoing findings potentially applicable there too; but further research is required to substantiate that conjecture. The recent introduction of mental health standards within the Quality and Outcomes Framework [29] which contributes to the payment of GPs in the UK has provided a further intervention at the contextual level. It remains to be seen – and researched – what impact this will have on normalisation of and improvement in the quality of mental health care provision in the primary care setting. Primary mental health care is also a setting in which clinical treatment and social care tend to be closely combined; where health problems are often chronic; where the evidence-base for interventions and models of care is frequently weak or absent; and where care is often provided through a fragmentary, networked implementation structure. These characteristics are also found elsewhere, for instance in healthcare for older people, for people with complex chronic physical health problems, and for the health care of offenders. Further research is required to substantiate the implication that the present findings apply to these domains too.

## Competing interests

The author(s) declare that they have no competing interests.

## Authors' contributions

RS led the clinical governance research team at NPCRDC. LG, RS and SC carried out the data collection for this part of the study. LG led the analysis of the data assisted by RS, AR and SC. LG drafted the manuscript which was read and approved by all the authors.

## Pre-publication history

The pre-publication history for this paper can be accessed here:


